# Supplementary material for: HERC1 Regulates Breast Cancer Cells Migration and Invasion
Source: Cancers (Basel). 2021 Mar 15;13(6):1309. doi: 10.3390/cancers13061309 (PMC8061768; doi:10.3390/cancers13061309)
Supplement: Supplementary file 1 [file cancers-13-01309-s001.zip › cancers-1116558-supplementary/figure_s1_herc1_validation_using_mdamb436cells.pdf]

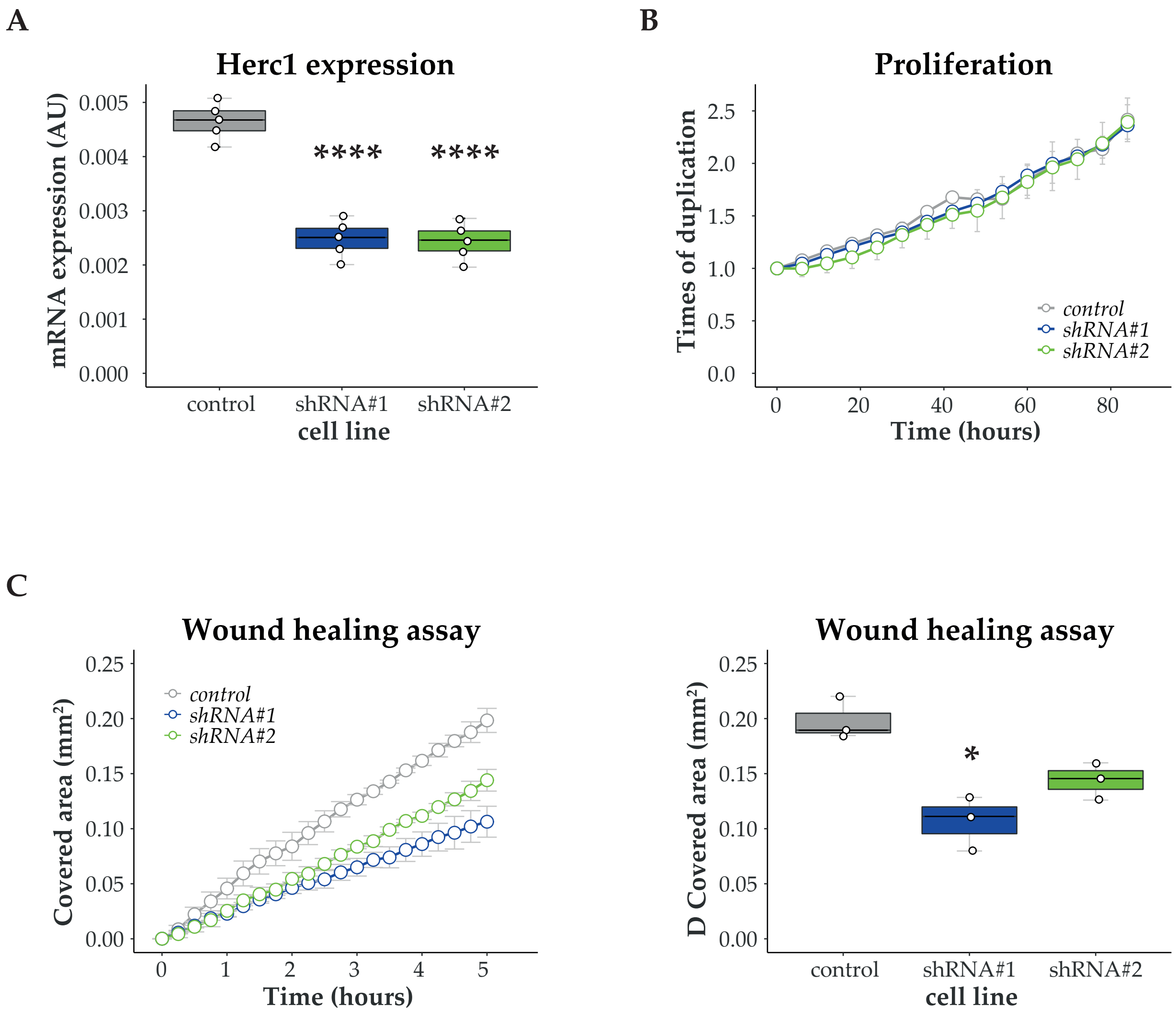

	control	shRNA#1	shRNA#2
speed: [mm/hr]	$0.028 \pm 5\times10^{-4}$	0.015 ± 0.002	$0.022 \pm 8\times10^{-4}$
p-value		0.0146	0.3594

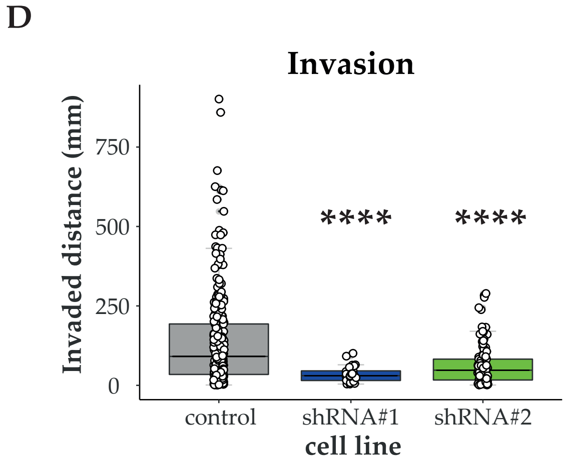

Figure S1| *In vitro* validation of HERC1 using MDA-MB-436 cells.

(**A**) Efficiencies of shRNA-mediated HERC1 knockdown were confirmed by RT-PCR (top, n= 5, one-way ANOVA, Dunnett's multiple comparison test. shRNA#1 $p< 0.0001$ and shRNA#2 $p< 0.0001$). (**B**) An area-based microscopy method was used to determine cell growth over time. Cells were seeded onto wells and allowed to attach. At the indicated time points, cells were photographed. The graph shows the occupied area relative to time= 0 hours (Doubling times= control: 67.91±1.485, shRNA#1 62.99±1.508, shRNA#2 75.25±8.893. n= 3, Kruskal-Wallis, Dunn's multiple comparison test. shRNA#1 $p= 0.5480$ and shRNA#2 $p= 0.2485$). (**C**) Wound healing assay was performed to analyze HERC1 silencing effects on MDA-MB-436 cells migration. Top left: wound covered area (mm^2) at the indicated timepoints. (n= 3, Extra sum-of-squares F Test. shRNA#1 $p< 0.0001$ and shRNA#2 $p< 0.0001$), bottom left: wound edge closing speed (n= 3, Kruskal-Wallis, Dunn's multiple comparison test. shRNA#1 $p= 0.0146$ and shRNA#2 $p= 0.3594$). Right: wound covered area (mm^2) at endpoint (n= 3, Kruskal-Wallis, Dunn's multiple comparison test. shRNA#1 $p= 0.0225$ and shRNA#2 $p= 0.2721$). (**D**) Agar spot assay was used to analyze invasion; the graph shows the cells displacement at the end of the experiment (n=1, more than 25 individual cells were measured per cell line. Kruskal-Wallis, Dunn's multiple comparison test. shRNA#1 $p< 0.0001$ and shRNA#2 $p< 0.0001$).
